# Tourette syndrome research highlights from 2018

**DOI:** 10.12688/f1000research.19542.1

**Published:** 2019-07-01

**Authors:** Olivia Rose, Andreas Hartmann, Yulia Worbe, Jeremiah M. Scharf, Kevin J. Black

**Affiliations:** 1 Department of Neuroscience, Washington University School of Medicine, St. Louis, MO, 63110, USA; 2 Sorbonne University, National Reference Centre for Tourette Disorder, Pitié-Salpêtrière Hospital, Paris, France; 3 Psychiatric and Neurodevelopmental Genetics Unit, Massachusetts General Hospital, Boston, Massachusetts, USA; 4 Psychiatry, Neurology, and Radiology, Washington University School of Medicine, St. Louis, MO, 63110, USA

**Keywords:** Tourette syndrome, tic disorders, review, natural history, etiology, pathophysiology

## Abstract

This is the fifth yearly article in the Tourette Syndrome Research Highlights series, summarizing research from 2018 relevant to Tourette syndrome and other tic disorders. The authors briefly summarize reports they consider most important or interesting. The highlights from 2019 article is being drafted on the Authorea online authoring platform, and readers are encouraged to add references or give feedback on our selections using the comments feature on that page. After the calendar year ends, the article is submitted as the annual update for the Tics collection on F1000Research.

## Introduction

This article is meant to disseminate recent scientific progress on Gilles de la Tourette Syndrome (TS).

## Methods

We searched PubMed from time to time during 2018 using the search strategy “(”Tic Disorders“[MeSH] OR Tourette NOT Tourette[AU]) AND 2018[PDAT] NOT 1950:2017[PDAT]”. On 20 May 2019 this search returned 192 citations. Colleagues also recommended articles, and we attended selected medical conferences. We selected material for this review subjectively, guided by our judgment of possible future impact on the field.

## Results

### Phenomenology and natural history

***Epidemiology.*** All chronic tic disorders (CTD) begin at some point in time; it has not been clear which children with a recent-onset tic disorder would go on to develop a chronic tic disorder, and which would permanently remit. Kim and colleagues report on 43 children initially studied an average of 3 months after tic onset, of whom 39 returned at the 12-month anniversary of their first tic ( Kim *et al*., 2018a; Kim *et al*., 2019). Not surprisingly, symptoms had generally improved at the 12-month mark. Surprisingly, however, every child still had tics, some apparent only when observed alone (by video). Baseline predictors of more severe tics at outcome included baseline tic severity, subsyndromal autism spectrum symptoms, three or more phonic tics, or an anxiety disorder. These results suggest that the current 12-month cutoff for chronicity may be arbitrary, and that mild tics that are not evident on a typical brief examination may be much more common than previously known.

Lowe and colleagues performed a very long interval follow-up study on TS, assessing tic severity, quality of life, and psychosocial function ( Lowe *et al*., 2019). Of 150 surveys mailed to patients seen 25–32 years ago, 45 were returned (30%). 79% reported still having at least some tics, 40% reported some level of social impairment, and 20% were either unemployed, disabled or financially supported by family. However, the great majority of patients had improved in terms of tics and were doing well in other areas of their lives. Tics are 4-6 times more common in children with attention deficit hyperactivity disorder (ADHD) than in those without, and tics add to clinical problems and reduced quality of life ( Poh *et al*., 2018). 

***Sensory phenomena and premonitory urges.*** Jakubovski *et al*. administered an online survey to characterize premonitory urges in individuals with TS ( Jakubovski *et al*., 2018). The authors found that premonitory urges occurred in 73% of their sample, and that premonitory urge was more likely to occur with complex tics (78.6%) over simple tics (68.9%). Premonitory urges tended to localize in the same body area as the tics themselves, contrary to previous, smaller reports.

Interoception is interesting as it shares features with sensory phenomena in TS ( Khalsa *et al*., 2018). A derived measure of interoception predicts tic severity and premonitory sensations in TS; the authors propose their results suggest “a heightened higher-order sensitivity to bodily sensations in TS, relative to a noisier perceptual representation of afferent bodily signals” ( Rae *et al*., 2019).

Impaired olfactory function, but normal peripheral detection of olfactory stimuli, was demonstrated in TS ( Kronenbuerger *et al*., 2018). Subjects with TS (n = 28) did not differ in odor detection threshold, but did present with impairments in both odor discrimination and odor identification. There was no significant difference in tobacco usage between the groups. 25% percent of subjects with TS met criteria for functional anosmia, compared to 7% of controls. This suggests that alterations in olfactory phenomena likely occur during higher-order processing, rather than during peripheral detection, in people with TS. This observation is consistent with previous similar conclusions about tactile sensory function in TS.

***Other.*** Weingarden *et al*. report that self-esteem is decreased in adults with TS and CTD (n = 122) ( Weingarden *et al*., 2018). This, however, is less related to tics or tic severity but rather related to other psychiatric symptoms. When treating these patients, self-esteem was improved more by Comprehensive Behavioral Intervention for Tics (CBIT) than by psychoeducation and supportive therapy (PST), which seems plausible. It would be interesting to replicate the same study in children and adolescents. A review also examines the concept of self-esteem in TS and CTD, drawing a similar conclusion: that poor self-esteem appears more strongly related to psychiatric comorbidities than to tic severity, and, unsurprisingly, affects quality of life ( Silvestri *et al*., 2018).

TS phenotypes were investigated in 174 children and adolescents in French university clinic ( Cravedi *et al*., 2018). Three clusters were identified. One of them corresponded to a tic-only phenotype (’pure’ TS) whereas another cluster included learning and intellectual disabilities, ASD, and ADHD. The third cluster corresponded to an ADHD profile with rather high intelligence and handwriting problems due to tics. Therapeutic implications are discussed.

A survey of almost 700 parents, about half with tics, found that children with current or past tics slept an average of 1.5 hours less per night than control children ( Ricketts *et al*., 2017). This result supports clinical experience and underlines both the impact of tic disorders on quality of life and a potential avenue for treatment. In children and adolescents with obsessive-compulsive disorder (OCD), religious symptoms were not associated with various clinical variables including outcome ( Wu *et al*., 2018). This result suggests that scrupulosity and similar symptoms in children with TS can be expected to improve just as much as other OCD symptoms with appropriate treatment.

While restricted food preferences have been well-documented in autism spectrum disorder (ASD), Smith *et al*. found similar patterns of food sensitivity in 30 children with TS. 25 subjects had comorbid conditions, though none in the sample had been diagnosed with ASD. Caregivers of children with TS reported their children having greater sensory sensitivities to food, greater food selectivity, and decreased consumption of dairy, vegetables, and fruit ( Robbins, 2018; Smith *et al*., 2019).

Severe neck tics led to a vertebral artery dissection ( Aydin *et al*., 2018), a reminder that tics are not always benign. Another case report describes closed head trauma due to head banging in a 15-year-old, with important sequelae ( Fasano & Galluccio, 2018). These reports underline the need for rapid and aggressive tic management in some patients. 

### Etiology

***Genetics.*** There have been a number of advances in TS genetics over the past year, each arising from large-scale, multi-consortia collaborative efforts. Building off of two articles published the previous year reporting an increased genome-wide significant burden of rare, gene-disrupting mutations in 5–10% of TS patients ( Huang *et al*., 2017; Willsey *et al*., 2017), Wang and colleagues reported the largest study of rare, *de novo* gene-disrupting mutations in TS to date ( Wang *et al*., 2018). After first demonstrating that this class of mutations ( *de novo* likely gene-damaging coding mutations and gene-disrupting copy-number variants/deletions) were specifically enriched in TS patients *without a family history of a tic disorder* (two unaffected parents) compared to patients with a positive parental history of tics, they conducted a combined *de novo* analysis of 802 parent-proband trios that identified a new high confidence TS susceptibility gene *CELSR3*, as well as an observation that the group potentially causative new mutations were more likely to affect genes involved in cell polarity. In parallel, Yu and colleagues reported the latest TS genome-wide association study (GWAS) of 4819 cases and 9488 controls, in which they identified one strong candidate TS gene ( *FLT3)*, and confirmed prior findings that a large proportion of TS heritability can be attributed to the aggregated effects of common genetic risk variants spread across the genome ( Yu *et al*., 2019). They subsequently demonstrated that, in subjects *with a family history of a tic disorder* (TS or chronic tics), aggregated genome-wide TS polygenic risk scores (PRS) were significantly associated with lifetime worst-ever tic severity scores. In addition, both Yu and colleagues and Abdulkadir and colleagues used the TS GWAS polygenic risk scores to probe two independent population-based GWAS samples and found that individuals with non-TS tic disorders also have elevated TS polygenic risk compared to unaffected controls, albeit to a lesser degree than individuals with TS ( Abdulkadir *et al*., 2019); ( Yu *et al*., 2019). These two findings therefore confirm at the genetic level that TS and other tic disorders likely exist along a continuous spectrum as opposed to their current classification as distinct diagnostic entities. Lastly, Mufford and colleagues used the same TS GWAS PRS to probe imaging genetic data of subcortical brain volumes in the ENIGMA (Enhancing Neuro Imaging Genetics Through Meta Analysis) consortium, essentially tying the genetics of TS to the genetics of brain volumes ( Mufford *et al*., 2018). 

Separately, a huge study of shared GWAS data from over a million people with various neurologic and psychiatric disorders revealed that TS shares common variant genetic risk with OCD, major depression, and, unexpectedly, with migraine, especially migraine with aura ( Brainstorm Consortium *et al*., 2018).

***Environmental risk factors.*** A large, full-population study from Sweden studied all singleton births in Sweden over a 30-year period, using siblings as controls ( Brander *et al*., 2018). It found that “impaired fetal growth, preterm birth, breech presentation and cesarean section were associated with a higher risk of” TS or CTD. The risks were dose-dependent, with hazard ratios rising from 1.41 for one adverse perinatal event to 2.42 for five or more such events. This report is important due to its careful design, sample size and implications. It confirms previous indications that not all of the risk for TS is inherited, and points specifically to intrauterine and birth insults as contributing to that risk.

A nationwide study from Denmark showed that children treated with an antibiotic or admitted to a hospital for an infection had a significantly higher risk of a later diagnosis of any psychiatric illness ( Köhler-Forsberg *et al*., 2019). Interestingly, the highest risk for antibiotic use was for tic disorders, followed by OCD. (Hospitalization risk was higher for intellectual disability, though tic disorders were next highest.) The association does not prove that infections cause TS; *e.g.*, patients destined to develop TS, or their parents, may be more likely to seek help for infections. Nevertheless, the association is interesting and deserves follow-up.

### Pathophysiology

***Animal models.*** Nespoli *et al*. (2018) found that dopaminergic imbalance in the dorsal striatum induced a Tourette’s-like phenotype in a rodent model. Administration of quinpirole, a selective D2/D3 receptor agonist, in juvenile rats with lesions to striatal projection neurons produced movements suggestive of both simple and complex tics in the neck, limbs, and mouth. A modified Yale Global Tic Severity Scale (YGTSS) was created to comprehensively score tic-like movements based on frequency, complexity, and severity of impairment. Immunohistochemical analyses revealed significantly decreased D1 receptor RNA expression at the lesion site, consistent with the decreased striatal D1 receptor expression seen in a human post-mortem study of TS ( Lennington *et al*., 2016). The dopaminergic imbalance induced by decreased striatal D1 receptor activity, coupled with increased D2 receptor activity, may be relevant to TS. 

***Electrophysiology.*** Eight awake TS patients undergoing DBS electrode implantation had recordings of individual cells of the external and internal globus pallidus (GP) ( Israelashvili *et al*., 2017). Some cells in each division of the GP showed transient changes in firing rates associated with tics.

***Neuroimaging studies.*** Mahone *et al*. utilized 7T spectroscopy to investigate glutamate and GABA concentrations in children with TS. Findings included increased premotor area glutamate concentrations in TS, which also positively correlated with inhibitory control ( Mahone *et al*., 2018).

White matter tractography in children and adolescents with TS has not been well characterized, as previous studies utilizing diffusion tensor imaging (DTI) have primarily sampled from adult TS patients. Sigurdsson *et al*. examined white matter connectivity and water diffusivity in youth with TS, and report widespread decreases in axial diffusivity ( Sigurdsson *et al*., 2018). The authors excluded subjects with high head motion, but even small head movements can affect DTI estimates ( Baum *et al*., 2018).

In an event-related Functional magnetic resonance imaging (fMRI) study of 15 adults with TS, successful tic suppression correlated with increased activation in dorsal anterior cingulate cortex ( van der Salm *et al*., 2018). 22 healthy controls performed a blink suppression task. During voluntary blink suppression, results yielded increased activation in ventrolateral prefrontal cortex, supplementary motor area, and cingulate motor area. These results suggest limbic system control of tic suppression, in contrast to sensorimotor system engagement in blink suppression. It would be an interesting follow-up to investigate whether suppression of normal blinks in TS patients displays the same patterns of activation seen in healthy controls, or reflects impairments in sensorimotor or limbic system control.

21 adults with TS and 21 healthy controls performed an fMRI study during perception of neutral or angry faces ( Rae *et al*., 2018). In TS, insula functional connectivity (fc) was increased with pre-supplementary motor area (SMA), premotor cortex, primary motor cortex and putamen. Insula fc with globus pallidus and thalamus varied with tic severity, while insula-SMA fc varied with premonitory sensations. These results strengthen the evidence that insula and SMA are involved with premonitory urges and tics.

***Pharmacological studies.*** Maia & Conceição (2018) elaborate on their theory of how dopamine may relate to tics in TS. Hienert *et al*. (2018) provide a meta-analysis of positron emission tomography (PET) and single-photon emission computed tomography (SPECT) studies in TS measuring the dopamine transporter (DAT) or D _2_-like dopamine receptors; neither showed conclusive differences in patients, but sample sizes and methodological limitations may explain the negative results.

***Clinical and neuropsychological studies.*** The potential role that autoimmunity may play in TS pathology has long been controversial. A large-scale population study from a Swedish birth cohort assessed the risk factors of 40 different autoimmune diseases in individuals with TS/CTD and/or OCD, as well as family members with varying degrees of relatedness ( Mataix-Cols *et al*., 2018). TS/CTD was associated with a 36% increase in likelihood of having any autoimmune disease, compared with matched population controls. Additionally, the mothers of subjects diagnosed with TS/CTD were 40% more likely to have an autoimmune disease; full siblings of individuals with TS/CTD carried a 17% increase in risk. The authors note that it is undetermined if these numbers support the hypothesized immunological component of TS/CTD, or instead reflect a selection bias for those already receiving medical care.

A research consortium in Germany has recently proposed that tics may correspond to altered perception-action binding ( Beste & Münchau, 2018). Petruo *et al*. (2018) now provide a first experimental demonstration in 35 adolescents with TS and 39 healthy controls using a Go/No-go task and subsequent electroencephalography (EEG) analysis, providing support for the idea that stimulus-action binding is stronger in patients with TS, and that “unbinding” may thus represent a useful therapeutic venue.

Inhibitory control in TS has been assessed in a myriad of different ways, with conflicting results. For instance, Mancini *et al*. (2018) report abnormal inhibitory function in patients with OCD, but no deficits in the TS group. Similarly, Stenner *et al*. (2018) performed a clever study of a form of automatic motor inhibition. Exposure to a subliminal stimulus at an appropriate time prior to a supraliminal movement cue inhibits movement, a phenomenon called the negative compatibility effect (NCE). Here, 21 adults with TS and 21 tic-free controls performed similarly, suggesting that NCE is another form of experimental motor inhibition that appears normal in TS.

By contrast, the role of abnormal habit-learning has been posited as a cognitive marker of TS. It is yet unclear if tics would result from increased habit-learning, deficits in unlearning maladaptive behaviors, or a combination of both. Shephard *et al*. (2018) investigated implicit sequence learning in youth with TS with or without ADHD compared to tic-free youth with or without ADHD. Subjects with ADHD demonstrated impaired learning of a motor sequence learning task, while subjects with TS had mildly enhanced learning. However, they found evidence of “hyper-learning,” in which subjects with TS had difficulty in unlearning the motor sequence task. Similarly, Kim *et al*. (2018b) demonstrated slower unlearning (forgetting) of a visuomotor task in children and adolescents with TS. The role that impaired habit unlearning may play in TS warrants further investigation.

Adaptive functioning, a person’s ability to function and organize themselves in daily life, has not been well-characterized in TS. Taylor *et al*. found that, in a TS sample, deficits in adaptive functioning were mostly driven by impaired executive function ( Taylor *et al*., 2018). ADHD and two or more comorbid conditions were associated with decreased adaptive functioning. More than half of the variance in this study was explained by deficits in executive function, rather than tics themselves. These results suggest that aggressive treatment of ADHD and comorbid diagnoses may be important in clinical management of TS.

### Treatment

***Psychological interventions.*** Group-based psychotherapeutic interventions for tics bear the promise of reduced costs and easier access to appropriate care, and several recent reports address this treatment option. One recent paper investigated the long term effects of group therapy on tic severity, quality of life and school attendance in 28 children with TS 12 months after completing habit reversal training (HRT) training or education (a follow up to the 2016 study ( Yates *et al*., 2016)). This study demonstrated positive effects in the long run but apparently without significant differences between both groups ( Dabrowski *et al*., 2018). A Danish study investigated a combined exposure and response prevention (ERP)/HRT protocol comparing group with individual sessions (n = 27 per group, n = 54 total). The efficacy on decreasing tic severity was similar in both treatment arms ( Nissen *et al*., 2019). Traditionally, ERP sessions (as compared to HRT/cognitive behavioral intervention for tics (CBIT)) lasted for two hours, making them more difficult and expensive. In this study, session duration was shortened to one hour and shorter exposure was as effective, if not more, than the classic format ( van de Griendt *et al*., 2018). In another potential method for wider behavior therapy accessibility, ( Andrén *et al*., 2019) report preliminary results from a major study on therapist-supported, parent-guided, online behavior therapy for TS. Patients improved substantially (mean 75% with ERP, 55% with HRT), and the improvement persisted up to 12 months later. The average therapist time was only 25 minutes per week.

***Medication.*** Swedish treatment registries were searched to identify patterns of medication prescribing for almost 7000 patients with TS/CTD from 2005–2013 ( Carulla-Roig *et al*., 2018). Among other interesting findings, ADHD drugs, antidepressants, and hypnotics/sedatives were all prescribed more often than antipsychotics, for which there is much stronger evidence of efficacy. Rizzo *et al*. (2018) provide the first direct comparison of pharmacotherapy with behavioral therapy in children and adolescents with TS / CTD. Both approaches were effective in reducing tics and improving quality of life; however, only pharmacotherapy was effective in reducing OC symptoms. 

The D1 receptor antagonist ecopipam was compared to placebo in a double-blind, crossover, randomized controlled trial (RCT) in children and adolescents with TS ( Gilbert *et al*., 2018). YGTSS total tic score (TTS) declined significantly more with the active drug. Another drug acting largely on dopamine, the VMAT2 inhibitor valbenazine, was tested in a phase IIb study in TS, but failed to meet the primary efficacy endpoint ( Neurocrine Biosciences Inc., 2018).

***Neurosurgery.*** A large-scale public database and registry for deep brain stimulation (DBS) Tourette syndrome has been established ( Martinez-Ramirez *et al*., 2018). This report summarizes information on 185 Tourette patients from 10 countries. Mean improvement in total YGTSS score was 40% at 6 months after *vs.* before surgery, and 45% at 12 months. The difference between stimulation sites (centromedian nucleus (CM-Pf), anterior globus pallidus interna (GPi), posterior GPi) was not statistically significant. About a third of patients had side effects, mostly related to stimulation not surgery. A large, collaborative group is using this database to investigate the optimal site for DBS via a 3D analysis ( Johnson *et al*., 2018). DBS in children remains controversial. Coulombe *et al*. (2018) review available data on DBS in “children” (ages 12-21), and Smeets *et al*. (2018) discuss ethical considerations regarding DBS in TS patients under the age of 18.

***Other treatment.*** In a pilot study, Behler *et al*. administered bilateral cathodal transcranial direct current stimulation (tDCS) over motor cortex in three patients with TS. While one patient displayed a 35% improvement in tic severity, the other two subjects experienced worsening of symptoms. However, all three patients did report a decrease in negative affect, as well as a mild increase in positive affect ( Behler *et al*., 2018).

### Tics, family and society

Himle *et al*. (2018) review how tics can affect the family and vice versa. Two large studies have now demonstrated clearly how much TS can hinder school performance. The first is a large study, conducted by the CDC via telephone survey, from the US. The authors found that TS (specifically, when compared to children without TS both with and without other neuropsychiatric conditions) has negative consequences for school performance ( Claussen *et al*., 2018). Subjects with moderate-to-severe TS (n = 97), when compared to those with milder symptoms (n = 203), were significantly more likely to require an individualized education plan (IEP). Furthermore, the prevalence of co-morbid psychiatric illness in TS is quite high; 80% of subjects with TS had at least one co-morbid neuropsychiatric disorder, compared to 18% of subjects without TS. The second study is from the population registry in Sweden, demonstrating academic underachievement in TS/CTD across all educational levels ( Pérez-Vigil *et al*., 2018). One limitation of this study is that it relies on those who are diagnosed by a physician, who we know are a proper subset of those with tics. One would not be surprised to learn that the factors that lead to care-seeking (like severity of symptoms, or other undiagnosed behavioral problems) may also themselves interfere with education. Commenting on this article, Hartmann and Delorme write, “Ticcing as such impairs attention, and attempts to supress tics at school or work makes things even worse. Teasing and bullying because of tics is a further contributing factor to attention problems. Thus, tics impair academic achievement in their own right, and comorbidities can only partially be blamed for this situation. … TS and CTD are not harmless neurological conditions but profoundly affect a person’s life trajectory (see also Mataix-Cols’ work on suicide in TS ( Fernández de la Cruz *et al*., 2017)). Take tics seriously!” ( Hartmann & Delorme, 2018).

### Additional reference sources

Several useful review articles appeared during 2018 ( Efron & Dale, 2018; Ganos *et al*., 2018; Garnaat *et al*., 2018; Hartmann & Worbe, 2018; Stern, 2018).

## Conclusions

The literature on TS is increasing rapidly. Fortunately, studies with larger sample sizes are becoming more common. Several simple but important questions remain to be answered in TS, including the following: Why do tics tend to start at ages 5–10? Why are they more common in boys? Why do they tend to improve during sleep? Why do tics usually improve in early adulthood? How accurately can we predict outcome for an individual patient? Which patients need which treatments? Is secondary prevention possible? Hopefully future studies will address these and other important issues.
