# Supplementary material for: Tourette syndrome research highlights from 2018
Source: F1000Res. 2019 Jul 1;8:988. [Version 1] doi: 10.12688/f1000research.19542.1 (PMC6719747; doi:10.12688/f1000research.19542.1)
Supplement: Peer review history

# Peer review: Reviewer response for version 1

**DOI:** 10.5256/f1000research.21424.r52244

**Published:** 2019-09-02

**Authors:** Marc Lavoie

**Affiliations:** 1 Département de neurosciences, Université de Montréal, Montreal, QC, Canada; 2 Department of Psychiatry and Addiction, Université de Montréal, Montréal, QC, Canada

This is a well selected review that covers most of the essential 2018 works on Tourette syndrome.

 One area that will probably have a big impact in the future, is the ability to predict which neurocognitive variables represents good predictors of clinical evolution in TS. Electrophysiological measures were useful to predict outcome, following a psychological intervention, in other psychiatric disorders, such as anxiety disorders, depression and obsessive-compulsory disorder (Krause et al., 2015; Burkhouse et al., 2016) ^1^^,^^2^, but has yet to be tested in TS. One article did just that last year. I suggest to add an article by Morand-Beaulieu and colleagues (2018) ^3^ that suggest that electrophysiological markers could offers new predictors for therapeutic outcome in TS and CTD patients with its high temporal precision to follow the stream of fast cognitive and motor processes. Morand-Beaulieu et al (2018) suggested that motor-related and slow cortical potentials could constitute electrophysiological markers of TS.

 These measures are related to SMA and basal ganglia functioning which is pretty consistent with neuroimaging and electrophysiological studies already reported in the current manuscript. I believe that this article could fit well in the ‘’clinical and neuropsychological studies’’ section or perhaps in the ‘psychological intervention’ section if you decide to integrate it.

I confirm that I have read this submission and believe that I have an appropriate level of expertise to confirm that it is of an acceptable scientific standard.

## References

[1] : Prediction of Treatment Outcome in Patients with Obsessive-Compulsive Disorder with Low-Resolution Brain Electromagnetic Tomography: A Prospective EEG Study. *Front Psychol*.2015;6: 10.3389/fpsyg.2015.01993 1993 10.3389/fpsyg.2015.01993 26834658PMC4722125

[2] : NEURAL REACTIVITY TO REWARD AS A PREDICTOR OF COGNITIVE BEHAVIORAL THERAPY RESPONSE IN ANXIETY AND DEPRESSION. *Depress Anxiety*.2016;33(4) : 10.1002/da.22482 281-8 10.1002/da.22482 27038409PMC4818973

[3] : Electrophysiological predictors of cognitive-behavioral therapy outcome in tic disorders. *J Psychiatr Res*.105: 10.1016/j.jpsychires.2018.08.020 113-122 10.1016/j.jpsychires.2018.08.020 30219560

# Peer review: Reviewer response for version 1

**DOI:** 10.5256/f1000research.21424.r50717

**Published:** 2019-07-15

**Authors:** Lorena Fernández de la Cruz

**Affiliations:** 1 Department of Clinical Neuroscience, Centre for Psychiatry Research, Karolinska Institutet, Stockholm, Sweden

This is yet again a good summary of the most relevant TS literature published in the past year (2018). The text is useful for researchers and clinicians in the TS and related fields, and in my opinion constitutes a nice educational piece. The included papers are overall well chosen and summarised. I only have a few minor comments, listed below.

 The authors have included in their review the paper by Wu *et al*. (2018) ^1^, which refers to the clinical characteristics and treatment outcomes of children and adolescents with OCD with and without religious symptoms. This article concludes that the presence of religious symptoms is not associated with OCD treatment outcomes. Based on this, the authors of the current review affirm that this suggests that scrupulosity and similar symptoms in children with TS can be expected to improve just as much as other OCD symptoms with appropriate treatment. While this may be true, I think this conclusion is long stretched based on the Wu *et al*. (2018) data, which actually does not refer to TS or tics at all. I therefore suggest to remove this paper from the review.

 When the authors mention the treatment study by Andrén *et al*. (2019) ^2^, I think it is important to mention that the percentages in the parenthesis in the sentence “Patients improved substantially (mean 75% with ERP, 55% with HRT)” actually refer to treatment responders, and not the percentage of patients that simply improved.

 Last sentence of the “Etiology” section: “the association is interesting and deserves follow-up”. This reads weird to me and I suggest to change the wording to “deserves to be further explored” or similar.

 Please check the manuscript for typos; there are quite a few. Some of them are listed below:

- “Phenomenology and natural history” section; “Sensory phenomena and premonitory urges”, third paragraph: the verb is missing in the sentence “alterations in olfactory phenomena ARE likely to occur…”
- “Phenomenology and natural history” section; “Other”, second paragraph: “in A French university clinic” instead of “in French university clinic.”
- “Pathophysiology” section; “Neuroimaging studies”, third paragraph: write “functional” in lower case.
- “Treatment” section; “Neurosurgery”: a registry for deep brain stimulation (DBS) FOR Tourette syndrome…”
- Note that the acronym ASD appears before it is spelled out, two paragraphs later (page 3).

I confirm that I have read this submission and believe that I have an appropriate level of expertise to confirm that it is of an acceptable scientific standard.

## References

[1] : Comparing OCD-affected youth with and without religious symptoms: Clinical profiles and treatment response. *Compr Psychiatry*.86: 10.1016/j.comppsych.2018.07.009 47-53 10.1016/j.comppsych.2018.07.009 30077806PMC6245548

[2] : Therapist-guided and parent-guided internet-delivered behaviour therapy for paediatric Tourette's disorder: a pilot randomised controlled trial with long-term follow-up. *BMJ Open*.2019;9(2) : 10.1136/bmjopen-2018-024685 e024685 10.1136/bmjopen-2018-024685 30772854PMC6398666
